# Application of Behavior Change Techniques and Rated Quality of Smoking Cessation Apps in China: Content Analysis

**DOI:** 10.2196/56296

**Published:** 2025-03-24

**Authors:** Qiumian Hong, Shuochi Wei, Hazizi Duoliken, Lefan Jin, Ning Zhang

**Affiliations:** 1 School of Public Health and the Second Affiliated Hospital, School of Medicine, Zhejiang University, No. 866 Yuhangtang Road, Hangzhou, 310058, China

**Keywords:** smoking cessation, behavior change techniques, mobile application, content analysis, China

## Abstract

**Background:**

Smoking cessation apps are increasingly being used to help smokers quit smoking. In China, whether behavioral science–based techniques are being incorporated into smoking cessation apps remains unknown.

**Objectives:**

This study aims to describe the usage of behavior change techniques (BCTs) among smoking cessation apps available in China and to evaluate the relationship between BCT utilization and the quality of available smoking cessation apps.

**Methods:**

We searched eligible smoking cessation apps twice on September 12 and October 4, 2022. We coded them with BCTs and assessed their quality by the Mobile App Rating Scale (MARS) and rating score in the App Store. We described the quality of each app (ie, engagement, function, esthetic, and information) and the BCTs used within it, as well as the amount and proportion of all BCTs used. Correlation analysis and linear regression analysis were used to assess the association between the number of BCTs used and the quality of apps.

**Results:**

Nine apps were included in the final analyses. The average number of BCTs being used was 11.44 (SD 2.57), ranging from 5 to 29. Only 1 app used more than 20 BCTs. The most frequently used BCTs were providing feedback on current smoking behavior (9/9, 100%), prompting review of goals (8/9, 88.89%), prompting self-monitoring of one’s smoking behavior (7/9, 77.78%), and assessing current and past smoking behavior (7/9, 77.78%). The most commonly used BCTS specifically focus on behavior, including BM (B refers to behavior change, M focuses on addressing motivation; 4.44/11, 40.36%) and BS (B refers to behavior change, S refers to maximizing self-regulatory capacity or skills; 3.78/11, 34.36%). The average score of MARS for the apps was 3.88 (SD 0.38), ranging from 3.29 to 4.46, which was positively correlated with the number of BCTs used (*r*=0.79; *P*=.01). Specifically, more usage of BCTs was associated with higher engagement score (β=.74; *P*=.02; *R*^2^=0.52) and higher information score (β=.76; *P*=.02; *R*^2^=0.52).

**Conclusions:**

The quality of smoking cessation apps assessed by MARS was correlated with the number of BCTs used. However, overall, the usage of BCTs was insufficient and imbalanced, and the apps demonstrated low quality of engagement and information dimensions. Coordinated efforts from policy makers, technology companies, health behavior professionals, and health care providers should be made to reduce tobacco consumption and to develop high-quality, widely accessible, and effective smoking cessation apps to help smokers quit smoking.

## Introduction

Tobacco smoking is one of the major public health challenges worldwide [1]. According to the World Health Organization (WHO) Western Pacific [2], the prevalence of smoking in the Chinese adult population was 28% in 2015. In addition, tobacco smoking is a major risk factor for many types of cancer, cardiovascular, and respiratory diseases [3-6], placing a heavy burden on both smokers and health care systems. Smoking cessation is an effective approach to preventing diseases related to smoking and reducing the risk of death due to smoking [7]. Traditional ways to increase smoking cessation mainly include providing advice, taking medicines such as bupropion and varenicline, carrying out cessation counseling, and organizing smoking cessation support groups [8,9].

With the development of mobile health, smoking cessation apps are increasingly being used to help smokers quit [10,11]. The convenience and accessibility of smoking cessation apps make it easier for smokers to monitor and change their smoking behaviors. In China, the widespread adoption of mobile technology, with about 1.1 billion mobile web users in 2024, has led to the rapid growth of mobile apps. Public awareness of health has increased, leading to greater adoption of health-related apps for services such as telemedicine and chronic disease management (300 billion in 2022 to 365 billion in 2024) [12]. There were several smoking cessation apps available in China [13]. Based on the previous studies, the function and the quality of these apps varied [13-15]. It is important to evaluate these apps with a scientific approach to help smokers choose the most useful and appropriate app and improve the design and development of smoking cessation apps.

The behavior change techniques (BCTs) developed by Michie and colleagues [16] provide a detailed taxonomy of the methods to change behaviors. During the past decade, BCTs have been increasingly used among mobile apps for promoting health behavior change. To explore the components that make a behavior change app effective and assess the reliability of the function, researchers coded behavior change strategies used in apps focused on health behaviors with BCTs, such as physical activity [17], dietary [18], alcohol consumption [19], and emotion management [20].

Furthermore, researchers investigated the impact of BCTs usage on app quality. A study aimed to recommend apps used for health management during pregnancy showed a positive and significant relationship between the number of BCTs used and the quality assessed with the Mobile App Rating Scale (MARS) [21]. Another research in France also demonstrated that the mean MARS score was positively correlated with the amount of BCTs used, but it also reported that fewer popular smoking cessation apps were developed based on BCTs [14]. There have been studies investigating the basic features (such as release date, frequency of downloads, user ratings, and so on) of smoking cessation apps in China [13,22]. However, no previous research has examined the use of BCTs and its relationship to app quality among smoking cessation apps in China. The study aims to fill this gap by describing the number and type of BCTs being used among smoking cessation apps in China and evaluating the relationship between BCT usage and app quality rated by MARS.

## Methods

### Study Design

We conducted a content analysis of the smoking cessation apps in China. First, one author searched Huawei App Market (Android) and App Store (iOS) twice by keywords (in Chinese, “戒烟,” “吸烟,” and “抽烟”) to initially identify relevant smoking cessation apps. Then, the final app list was selected by 2 researchers (SW and LJ) based on the inclusion and exclusion criteria. Next, 2 researchers independently coded usage of BCTs in the smoking cessation apps based on a taxonomy of smoking cessation BCTs [23] and rated their quality according to the MARS. Two researchers (SW and LJ) had a moderate to high levels of agreement (κ=0.67) on coding the apps. For inconsistent codings, we discussed and consulted the opinion of the corresponding author to reach a consensus for the final coding.

### Search Strategy and Data Extraction

According to the latest report from AppInChina, Huawei App Market was the most used Android App Store in China, with a market share of 44.31%. Considering the market share of iPhone in the Chinese smartphone market is 15%‐20%, we searched both on the Huawei App Market (Android) and App Store (iOS). One researcher (SW) searched twice on the 2 app stores on September 12 and October 4, 2022, to make sure that all apps were included. The inclusion criteria were as follows: (1) the app’s aim was to help users quit smoking, and (2) it was available for free download. A total of 69 apps were found. Meanwhile, the researcher collected the rating score (1‐5 points), number of downloads for Android, and number of ratings for all iOS apps. Then, 2 researchers (SW and LJ) downloaded and tested these apps. Finally, 9 apps were included based on the inclusion criteria. The exclusion criteria were apps that (1) did not support Chinese or did not have a Chinese version, (2) were not designed for smoking cessation only, (3) were irrelevant to smoking cessation, (4) were unable to use because of parameter problems or version incompatibility, and (5) had <10,000 downloads for Android or <50 raters for iOS. The reason for the different criteria for Android and iOS is that the Huawei App Market (Android) and App Store (iOS) have different popularity indicators.

### BCTs Coding

We adopted the 43-item taxonomy of BCT for smoking cessation developed by Michie et al [16], which was widely used in studies on smoking cessation interventions [23]. The taxonomy identified 43 BCTs and these techniques could be classified into six functions: (1) directly addressing motivation (BM; B refers to behavior change, M focuses on addressing motivation), (2) maximizing self-regulatory capacity or skills (BS; B refers to behavior change, S refers to self-regulatory capacity or skills), (3) promoting adjuvant activities (A), (4) supporting other BCTs (RD; R relates to general aspects of the interaction, D refers to development of intervention), (5) supporting other BCTs (RI; R relates to general aspects of the interaction, I refers to information gathering), and (6) supporting other BCTs (RC; R relates to general aspects of the interaction, C refers to general communication).

### MARS Rating

We adopted the MARS to assess the quality of the apps [24]. This 5-point scale was developed to assess the mHealth app quality with the following subscales: Engagement, Functionality, Esthetics, Information, and a subjective subscale. In reference to previous studies with good consistency [25,26], an overall quality score was calculated by taking the mean of items from the Engagement, Functionality, Esthetics, and Information subscales. Two researchers rated the included smoking apps using this scale together and had a high interclass correlation coefficient (0.85).

### Statistical Analysis

The interrater reliability was assessed by interclass correlation coefficient (for MARS) and kappa (for BCT coding). Descriptive analysis was used to describe the characteristics of the apps, including the overall, categorized, and individual BCT usage, as well as the app’s quality (MARS and user-rating scores). Correlation analysis was adopted to assess the relationship between the number of BCTs and the quality of apps (including MARS score and user rating in the App Store). Linear regression analysis was used to investigate whether the number of BCTs used was a significant predictor of the total score and the scores of each dimension of MARS. All the analyses were conducted using SPSS 19.0 (IBM Corporation), with a significance level of α=.05.

## Results

### Overview

**Figure 1.**
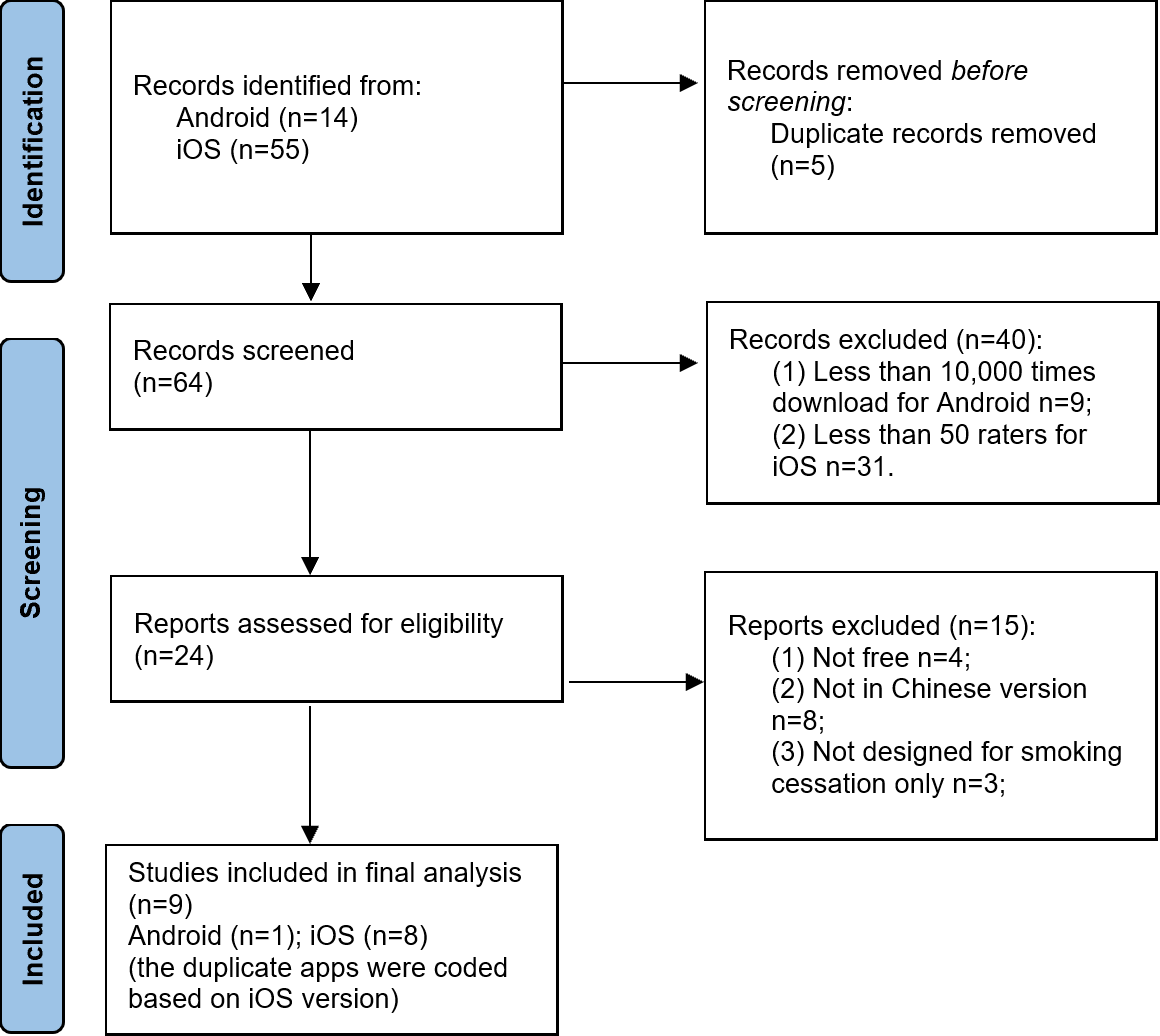
App screening flowchart.

A total of 69 apps were found after 2 rounds of searching. Of them, 20.29% (14/69) were developed for the Android system, 79.71% (55/69) of apps were developed for the iOS system, and 7.24% (5/69) of apps were excluded as they were duplicates in both systems. Of the remaining 64 apps, 55 apps were excluded based on the exclusion criteria (Figure 1), and finally, a total of 9 apps were coded. Detailed information for each app is shown in Table 1.

**Table 1.** Detailed information of each app (n=9).

	App name^a^	Number of BCTs^b^ used	MARS^c^ score					User rating	Number of raters	File size (MB)
			Engagement	Functionality	Esthetics	Information	Total			
1	JYJT	29	4.50	4.75	4.50	4.08	4.46	4.50	2016	24.4
2	WY	16	3.70	4.88	4.50	3.67	4.19	4.80	111	13.5
3	XZJY	14	3.20	4.88	4.17	3.42	3.91	4.90	336	77.2
4	JYYS	12	3.30	4.88	3.67	3.92	3.94	4.80	593	39.3
5	JY	9	3.80	5.00	4.33	3.67	4.20	4.90	309	3.2
6	JRCY	7	3.90	4.25	4.17	3.17	3.87	4.70	120,000	88.3
7	JRJY	6	2.50	4.00	3.50	3.17	3.29	4.80	546	2.5
8	CYYS	5	2.80	4.75	3.83	3.46	3.71	4.70	525	101.7
9	CYJL	5	2.20	4.88	3.00	3.42	3.37	4.60	30,000	16.3

a The app name is the pinyin initials of the Chinese name.

b BCT: behavior change technique.

c MARS: Mobile App Rating Scale.

### Usage of BCTs in Smoking Cessation Apps

As shown in Table 2, the average number of BCTs used by the 9 apps was 11.44 (SD 2.57), ranging from 5 to 29. Among the apps, 1 app that used the most BCTs used 29 BCTs, accounting for 67.44% of all BCTs, while 2 apps that used the least amount used only 5 BCTs, accounting for 11.63% of the BCTs for smoking cessation. The items in the BM (focusing on behavior change and addressing motivation) category were the most used BCTs in the apps (4.44/11, 40.36%), followed by the items in the BS (focusing on behavior change and self-regulatory capacity or skills) category (3.78/11, 34.36%). The least used items were those in the interaction category, focusing on the RD aspect (0.22/2, 11%).

**Table 2.** Average usage of behavior change techniques in smoking cessation apps in China.

Item	Label	Mean (SD)	Percentage (mean/total)
Average	The average number of BCT^a^	11.44 (2.57)	26.60 (11.44/43)
BM	Specific focus on behavior (B) and addressing motivation (M)	4.44 (2.60)	40.36 (4.44/11)
BS	Specific focus on behavior (B) and maximizing self-regulatory capacity or skills (S)	3.78 (2.82)	34.36 (3.78/11)
A	Promote adjuvant activities (A)	1.00 (1.11)	20 (1/5)
RD	General aspects of the interaction (R) focusing on delivery of the intervention (D)	0.22 (0.44)	11 (0.22/2)
RI	General aspects of the interaction (R) focusing on information gathering (I)	0.78 (0.44)	19.5 (0.78/4)
RC	General aspects of the interaction (R) focusing on general communication (C)	1.22 (1.79)	12.2 (1.22/10)

a BCT: behavior change technique.

The use of each BCT item is shown in Table 3. The most-used BCTs were BM3 (provide feedback on current behavior), BS5 (prompt review of goals), BS6 (prompt self-recording), RI1 (assess current and past smoking behavior), BM2 (boost motivation and self-efficacy), and BM6 (prompt commitment from the client there and then).

**Table 3.** Behavior change techniques (BCTs) used in each app.

BCT item	Label	App name^a^									Total (n=9)	Percentage
		JYJT (n=29)	WY (n=16)	XZJY (n=14)	JYYS (n=12)	JY (n=9)	JRCY (n=7)	JRJY (n=6)	CYYS (n=5)	CYJL (n=5)		
BM3	Provide feedback on current behavior	1	1	1	1	1	1	1	1	1	9	100
BS5	Prompt review of goals	1	1	1	1	1	0	1	1	1	8	88.89
BS6	Prompt self-recording	1	1	1	0	1	1	1	0	1	7	77.78
RI1	Assess current and past smoking behavior	1	1	1	1	1	1	0	0	1	7	77.78
BM2	Boost motivation and self-efficacy	1	1	1	1	1	0	1	0	0	6	66.67
BM6	Prompt commitment from the client there and then	1	1	1	1	1	0	0	1	0	6	66.67
BS4	Facilitate goal setting	1	1	0	1	1	0	1	0	0	5	55.56
BS9	Set graded tasks	1	1	0	1	1	0	1	0	0	5	55.56
BM1	Provide information on consequences of smoking and smoking cessation	1	1	0	1	0	0	0	0	1	4	44.44
BM7	Provide rewards contingent on effort or progress	1	1	1	1	0	0	0	0	0	4	44.44
BS3	Facilitate action planning/develop treatment plan	1	1	0	1	1	0	0	0	0	4	44.44
A2	Advise on/facilitate use of social support	1	1	1	0	0	1	0	0	0	4	44.44
A5	Give options for additional and later support	1	1	1	0	0	0	0	1	0	4	44.44
BM4	Provide rewards contingent on successfully stopping smoking	1	1	1	0	0	0	0	0	0	3	33.33
BM5	Provide normative information about others’ behavior and experiences	1	0	1	0	0	1	0	0	0	3	33.33
RC1	Build general rapport	1	1	0	0	0	1	0	0	0	3	33.33
RC8	Elicit client views	1	0	1	0	0	1	0	0	0	3	33.33
BM8	Strengthen ex-smoker identity	1	0	0	0	0	0	0	1	0	2	22.22
BM9	Identify reasons for wanting and not wanting to stop smoking	1	0	0	1	0	0	0	0	0	2	22.22
RD2	Emphasize choice	1	1	0	0	0	0	0	0	0	2	22.22
RC5	Offer/direct toward appropriate written materials	1	0	1	0	0	0	0	0	0	2	22.22
BM10	Explain the importance of abrupt cessation	0	0	0	1	0	0	0	0	0	1	11.11
BS1	Facilitate barrier identification and problem solving	1	0	0	0	0	0	0	0	0	1	11.11
BS2	Facilitate relapse prevention and coping	1	0	0	0	0	0	0	0	0	1	11.11
BS7	Advise on changing routine	1	0	0	0	0	0	0	0	0	1	11.11
BS8	Advise on environmental restructuring	1	0	0	0	0	0	0	0	0	1	11.11
BS11	Advise on avoiding social cues for smoking	1	0	0	0	0	0	0	0	0	1	11.11
A1	Advise on stop-smoking medication	1	0	0	0	0	0	0	0	0	1	11.11
RC6	Provide information on withdrawal symptoms	1	0	0	0	0	0	0	0	0	1	11.11
RC9	Summarize information/confirm on client decisions	0	0	1	0	0	0	0	0	0	1	11.11
RC10	Provide reassurance	1	0	0	0	0	0	0	0	0	1	11.11

a The app name is the pinyin initials of the Chinese name.

### General Quality of the Apps

The quality of apps was assessed by MARS and the rating score in the App Store. The average rating score of the 9 apps in the App Store was 4.74 (SD 0.13) with a range from 4.50 to 4.90. The average score of MARS was 3.88 (0.38) with a range from 3.29 to 4.46. The average scores of Engagement, Functionality, Esthetics, and Information were 3.32 (SD 0.73) (ranging from 2.20 to 4.50), 4.69 (SD 0.33) (ranging from 4.00 to 5.00), 3.96 (SD 0.51) (ranging from 3.00 to 4.50), and 3.55 (SD 0.31) (ranging from 3.17 to 4.08), respectively.

### Relationship Between Usage of BCTs and App Quality

**Figure 2.**
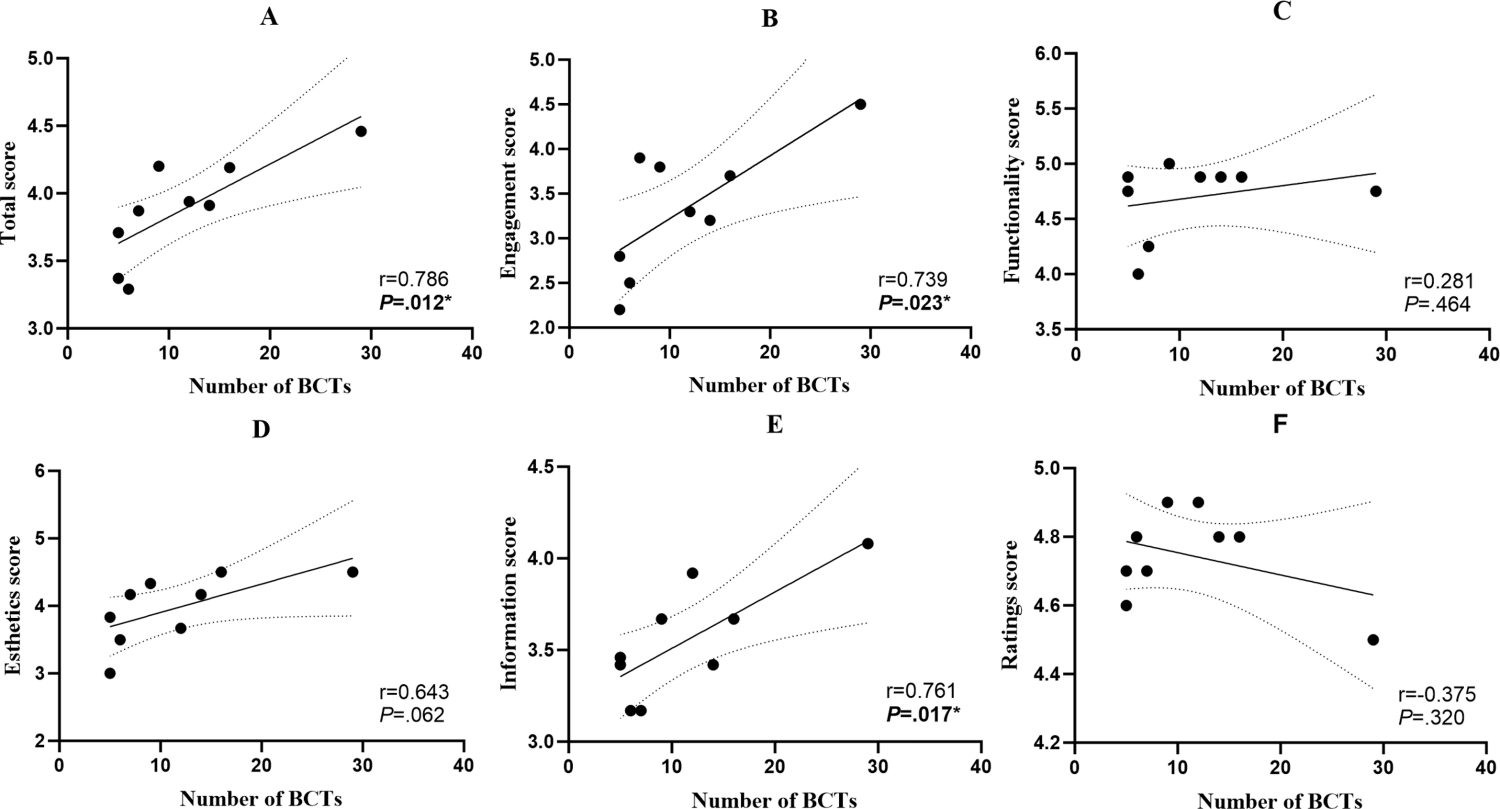
Correlation analysis between the number of BCTs and (A) the total score, (B) Engagement score, (C) Function score, (D) Esthetics score, (E) Information score, and (F) Rating score of apps. * *P*<.05. BCT: behavior change technique.

The results of the correlation analysis are shown in Figure 2. The number of BCTs was positively correlated with the total score of MARS (Figure 2A; *r*=0.786; *P*<.05) and the subscores of Engagement (Figure 2B; *r*=0.739; *P*<.05) and Information (Figure 2E; *r*=0.761; *P*<.05). There was no significant relationship between the number of BCTs being used and Functionality (Figure 2C; *r*=0.281; *P*=.46) or Esthetics (Figure 2D; *r*=0.643; *P*=.06) dimension of the apps. Besides, there was no significant relationship between the number of BCTs being used and the rating score (Figure 2F; *r*=0.375; *P*=.32).

For further verification, we conducted univariate linear regression analysis with the number of BCTs used as the independent variable and the MARS total score, the Engagement score, and the Information score as dependent variables. The results showed that the number of BCTs used in each app significantly predicted its MARS total score (β=.79; *P*=.01; adjusted *R*^2^=0.56; *F*_1, 7_=11.31, *P*=.01), its Engagement score (β=.74, *P*=.02; adjusted *R*^2^=0.48; *F*_1, 7_=8.40, *P*=.02), and its Information score (β=.76; *P*=.02; adjusted *R*^2^=0.52; *F*_1, 7_=9.76, *P*=.02). The amount of BCT used explained 56%, 48%, and 52% of the variation in the MARS total score, the Engagement score, and the Information score, respectively.

## Discussion

### Principal Findings

To our best knowledge, this was the first study to evaluate the appliance of BCTs and the quality of smoking cessation apps in China. The main findings were as follows: (1) the average number of BCTs used among smoking cessation apps was 11.44, with a range from 5 to 29; (2) the average score of MARS was 3.88, with a range from 3.29 to 4.46; and (3) the number of BCTs was positively correlated with the quality of apps assessed by MARS but not significantly associated with rating scores in App Stores.

The average number of BCTs being used in Chinese smoking cessation apps was 11.44 (SD 2.57), which was lower than that used among smoking cessation apps in other countries. A study reported that the mean number of BCTs was 22 in the French smoking cessation apps [14], which adopted the same BCT taxonomy in its coding process. In the detailed analysis, the included apps contained the BCTs that were mostly in the category of behavior and addressing motivation (BM) and behavior and maximizing self-regulatory capacity or skills (BS), which was in line with the characteristics of mobile apps. App is for users to download and operate independently; therefore, the focus of app design is to stimulate users’ motivation and enhance their self-management ability or skills. However, the most used BCT items served only to remind smokers to quit smoking and record smoking behaviors and lacked the assessment of smoking cessation status, explanation of smoking cessation behaviors, or communication and interaction with users, which can be integrated into app functions in the future. Furthermore, it is possible to evaluate the effect of these functions on improving the quality of apps and even increasing the smoking cessation rate of users.

As our results showed, the general quality of smoking cessation apps was not as high as assessed by MARS. The engagement score was the lowest, which indicated that smoking cessation apps in China may lack promotion or appeal. When the researchers rated the apps, they also found that most apps were not interesting or attractive enough to use, which could affect users’ engagement and stickiness. In future developments of smoking cessation apps, integrating personalized content and social features such as web-based communities and chat groups can help users feel that the app better meets their social needs and enhances their emotional attachment with the app. Gamification is also an effective way to increase engagement with apps [27,28]. Meanwhile, the information component scored second lowest among all the rated dimensions, indicating a lack of professional information and adequate data on smoking cessation (the benefits of quitting, potential obstacles and coping strategies, and information about withdrawal symptoms). It is worth noting that none of the apps we coded were evidence-based or validated by evidence or research. Researchers reported similar results when they coded the apps in France [14]. Notably, the relatively high functionality score may be due to the fact that most of the included apps have basic smoking cessation functions and are concise and smooth to use, meeting the scoring rules of this dimension.

We found that there was a positive relationship between the usage of BCTs and the quality of the apps as assessed by MARS. As hypothesized, the more BCTs being used, the higher the quality of smoking cessation apps. Specifically, the number of BCTs being used was significantly positively correlated with the engagement and information score of the apps. According to the MARS scoring rules, the use of BCTs may play a role in improving the pertinence, interactivity, and attractiveness of apps, and may provide guidance for the design of smoking cessation apps. However, we did not find a significant relationship between the usage of BCTs and rating scores in the App Store. For example, JY and XZJY were the 2 highest rating (4.9/5.0) apps but had lower usage of BCTs, and conversely, JYJT got the lowest rating (4.5/5.0) but used the most BCTs (29) and was rated with the highest MARS score (4.46). This finding suggested that ratings in stores were not sufficient indicators of app quality for smokers to choose quitting apps. Thus, to assist smokers select better apps, the App Store rating system should be more detailed, requiring users to rate different aspects of the app, such as functionality, information, and user engagement. In addition, users should compare multiple apps to find the one that best suits their needs and provide specific, timely feedback, such as leaving comments on the app.

Compared with previous studies, the number of smoking cessation apps in China was relatively small. There was a total of 69 apps available in the Huawei App Market (Android) and App Store (iOS), but only 9 apps met the inclusion criteria. A study from the United States searched 273 smoking cessation apps and analyzed 225 of them [29]. Another study from Australia searched 315 apps and analyzed 112 of them [26]. A team from the United Kingdom analyzed 140 apps [30], and a study from France searched 107 apps and analyzed 14 of them [14]. A recent study from Korea finally included 104 apps in analysis [15]. The difference in the number of smoking cessation apps revealed the big gap in the concept and market for smoking cessation or tobacco control between China and other countries. In fact, there is a growing number of studies on developing effective and scalable smoking cessation apps [31]. The behavior change theory, cognitive behavior therapy, and mindfulness training were the most frequently used theories in app development [32-34]. An app was developed under the guidance of BCTs for pregnant smokers [34]. Another app for smoking cessation was developed under the Behavioral Change Taxonomy version 1 [35]. Some apps were designed according to the mindfulness-based intervention [32], and others were designed based on cognitive behavioral therapy [33]. Several Chinese smoking cessation apps were developed using cognitive-behavioral theory [36] and protection motivation theory [37], and studies have demonstrated the feasibility and acceptability of the smartphone app intervention for smoking cessation [22]. However, we did not find them in the App Store, which indicated the need to translate the scientific evidence into public health practice.

In China, the development and promotion of health apps have seen significant progress. The government has actively supported the “Healthy China 2030” initiative [38], providing policy and financial backing for health app innovation. Furthermore, integration with telemedicine services, electronic health records, and artificial intelligence model has improved access to professional advice and streamlined patient care [12]. However, the frequency of use for health-related apps is much lower than that for communication, video, and food delivery apps. Moreover, although the usage of mobile health apps is increasing, there are demographic differences. Younger people and urban residents are more likely to use these apps than older adults and rural populations. Meanwhile, data privacy concerns, inconsistent app quality, a lack of collaboration with professional health care institutions, a lack of standardized regulations, and low user retention still exist and need urgent resolution. According to a report by WHO Western Pacific [2], there is the largest amount of tobacco consumption and number of smokers in China. However, the findings suggested that there were relatively few high-quality smoking cessation apps to help smokers quit. Coordinated efforts from policy makers, technology companies, and health care providers should be made to reduce tobacco consumption and to develop high-quality, widely accessible, and effective smoking cessation apps to help smokers quit in China.

### Limitations

There were some limitations in this study. First, the smoking cessation app market is changing and evolving, so this study can reflect only the status of smoking cessation apps so far. With the update and improvement of the app, we believe that the development of smoking cessation apps will be greatly improved. Second, we coded only the presence of the BCTs but did not evaluate or compare the design and presentation forms of the BCTs in each app, nor did we assess the actual usage of each BCTs by users of the smoking cessation apps. These factors were also important influences on the quality of the apps. In the future, researchers could analyze the actual use of BCTs and the differences between initial design and subsequent usage among smokers. Third, since fewer apps were eventually included in the analysis under strict inclusion and exclusion criteria, regression analysis could not be conducted to explore the effect of using a certain strategy on the app quality. Fourth, using the scores in the App Store as an evaluation metric to explore the relationship between app quality and BCTs usage may yield less accurate results. Ratings may come from individuals who have not fully experienced the app, leading to inaccuracies. Commercial influences can skew ratings, causing them to misrepresent the app’s true quality. Furthermore, a single flaw or standout feature can disproportionately affect the overall rating, making it an imperfect indicator of the app’s overall quality. Finally, the taxonomy of the BCTs we adopted was not specially designed for the mobile health app evaluation but instead used a taxonomy for smoking cessation. In this case, some of the items do not apply to apps, such as “measuring CO” and “Adopt appropriate local procedures to enable clients to obtain free medication.” Future research could develop BCTs that are more appropriate for the design and evaluation of smoking cessation apps.

### Conclusions

We searched and screened smoking cessation apps in China and evaluated the BCTs application and quality of the apps. We found that the BCTs were insufficiently used and the quality of the smoking cessation apps in China was generally low. This study provided a snapshot of the smoking cessation apps in China. There is an urgent need to design and develop more effective, attractive, and scalable smoking cessation apps to help smokers quit smoking.

## Abbreviations

BCT: behavior change technique
BM: specific focus on behavior and addressing motivation
BS: specific focus on behavior and maximizing self-regulatory capacity or skills
MARS: Mobile App Rating Scale
RC: general aspects of the interaction focusing on general communication
RD: general aspects of the interaction focusing on delivery of the intervention
RI: general aspects of the interaction focusing on information gathering
WHO: World Health Organization
